# Saving energy in residential buildings: the role of energy pricing

**DOI:** 10.1007/s10584-021-03164-3

**Published:** 2021-07-21

**Authors:** Jens Ewald, Thomas Sterner, Eoin Ó Broin, Érika Mata

**Affiliations:** 1 Department of Economics, School of Business, Economics and Law, University of Gothenburg, Vasagatan 1, 41124 Gothenburg, Sweden; 2 Centre International de Recherche sur l’Environnement et le Développement (CIRED), 45 Avenue de la Belle Gabrielle, 94736 Nogent-sur-Marne Cedex, France; 3 Swedish Environmental Research Institute (IVL), Aschebergsgatan 44, 41133 Gothenburg, Sweden

**Keywords:** Residential energy demand, Space heating, Energy efficiency, Buildings, energy, and climate policy, Carbon prices

## Abstract

A zero-carbon society requires dramatic change everywhere including in buildings, a large and politically sensitive sector. Technical possibilities exist but implementation is slow. Policies include many hard-to-evaluate regulations and may suffer from rebound mechanisms. We use dynamic econometric analysis of European macro data for the period 1990–2018 to systematically examine the importance of changes in energy prices and income on residential energy demand. We find a long-run price elasticity of −0.5. The total long-run income elasticity is around 0.9, but if we control for the increase in income that goes towards larger homes and other factors, the income elasticity is 0.2. These findings have practical implications for climate policy and the EU buildings and energy policy framework.

**Supplementary Information:**

The online version contains supplementary material available at 10.1007/s10584-021-03164-3.

## Introduction

Climate change has become a major focus of European policymaking. Goals have been ratcheted up to more ambitious targets but at the same time, the EU and individual countries like Germany have expressed concerns that progress in the reduction of carbon emissions from the ESR (Effort Sharing Mechanism) sectors such as transport and buildings is too slow (IEA 2020). Across the continent, buildings are the largest energy-consuming sector (40% in the EU), ahead of transport and industry.

A fossil-free society requires transformative change across all sectors—including in buildings, which account for 36% of greenhouse gas emissions in the EU (EC 2018). For many reasons, buildings need to be cornerstones of climate policy (Ürge-Vorsatz et al. 2020; Mata et al. 2020; Levesque et al. 2021). Technical solutions include super-efficient “zero-energy” houses even in semi-arctic areas (Niskanen 2018; Cabeza and Chàfer 2020). The reality, however, is millions of buildings waste energy and provide inadequate comfort—even in mild climates. Current policies in this sector consist of a myriad of detailed regulations concerning, e.g., insulation or window glazing but it is often hard to judge their effectiveness (Ó Broin et al. 2019a; Thonipara et al. 2019). When energy is cheap, the incentive to be consistent in saving energy is weak and thus pricing policy can be an important complement to existing policies. However, there is a hesitancy on the part of policymakers to use pricing policies since heating costs can have severe consequences on some low-income households and exacerbate incidences of fuel poverty.

Electricity production is in the midst of a renewable’s revolution (Mac Domhnaill and Ryan 2020) and fossil emissions from industry are also decreasing (Enerdata 2021a). Forecasts for the transport sector suggest there will be large-scale electrification which will reduce total demand at least in the mid to longer term (EEA 2017). Decreases in these sectors imply, unless action is taken, that the emissions share of the buildings sector increases. The European Commission recognizes these trends and highlights improvements in building efficiency in the European Green Deal (EC 2019) and other instruments as key to meeting the EU targets. These include the 2030 Climate and Energy Framework and the 2030 Climate Target Plan to cut greenhouse gas emissions by at least 55%. One new ambitious EU initiative is the Renovation Wave for Europe that combines COVID-19 recovery stimulus with investments in green renovation of buildings (EC 2020). Another is the Energy Performance of Buildings Directive that has required all new buildings from 2021 and onwards to be “nearly zero-energy” (EC 2018, 2021). EU countries also have additional domestic instruments such as efficiency standards for buildings and performance standards for appliances to reduce inefficient energy consumption. Although standards are harmonized for appliances that are traded on multiple markets, the same is not true for buildings where practices still vary by country. However, as all new constructions will be designed to be more energy-efficient, most of the variation is found in the existing building stock. For some efficiency measures, we may also expect rebound effects. Economic incentives, such as carbon or energy taxes, may mitigate rebound effects and reinforce the effect of regulations. In this context, it is crucial to understand the sensitivity of energy demand to fundamental economic variables such as personal income and energy prices.

Pricing policies are important since prices determine both the quantity and type of heating fuel used, as well as affecting intermediary variables such as indoor temperature, building insulation, maintenance, and even the floor area of a dwelling that is heated. The determinants of energy demand are often summarized by the price and income elasticities as the main drivers of change in demand for a good. The literature on fuel demand for *transport* numbers thousands of articles (Graham and Glaister 2004; Dahl 2012; Labandeira et al. 2017). There is also a wealth of literature on the technical determinants of energy demand as well as the economics of energy efficiency in the buildings sector (see Section 2). There are however fewer studies that focus on our main research issue: the price and income elasticities of residential energy demand. These elasticities are of critical importance for policy planning. In particular, our objective is to estimate reliable and up-to-date estimates of short- and long-run price and income elasticities for the EU residential sector by employing dynamic panel data estimators. As we motivate in Section 2, we believe we are the first to comprehensively study how energy prices have affected total residential energy use in all 27 EU countries and the UK at the macro level by using dynamic panel data econometrics.

## Literature

The literature on energy use in buildings is broad and includes different methodologies, perspectives, and fields of research (Cabeza et al. 2020; Mata et al. 2021). Recent surveys of the literature on determinants of buildings’ energy demand and greenhouse gas emissions show that the vast majority of the studies focus on electricity demand in buildings (Laes et al. 2018; Mata et al. 2021), whereas, in the EU, space heating demand accounts for over 65% and is driven by rather different factors than those that drive electricity demand for appliances (Unander et al. 2004; Brounen et al. 2012).

Micro-level studies that use data at the household level have contributed many important insights, not least in terms of variations across household characteristics. For instance, Brounen et al. (2012) utilize data from more than 300,000 homes in the Netherlands and show that electricity use varies with family composition and income levels, while Harold et al. (2015) identifies socio-economic characteristics and local climate as important determinants. Salari and Javid (2017), by analyzing a rich US dataset, find that among other determinants, demographical features and building characteristics are the two most important determinants of household energy expenditure, similar to some of the findings by Kavousian et al. (2015) that studies Irish household data. Further evidence of influences on energy demand from household characteristics have been observed in a range of other countries (Couture et al. 2012 in France; Ajayi 2018 in Nigeria; Jayaraj et al. 2011 in India; Tilov et al. 2019 in Switzerland; and Bissiri et al. 2019 who compares German and British households). Other micro studies that focus on more classical economic determinants of energy demand have found a negative relationship with energy price (Filippini 2011) and a positive relationship with income (as well as education; Hansen 2016). There are also micro studies of the important notion that ownership form can influence demand (Meier et al. 2013; Kavousian et al. 2013, 2015; Ahmed et al. 2013; Engvall et al. 2014; Harold et al. 2015; Hung and Huang 2015).

In addition to the abovementioned studies, there is a large micro-level literature on the so-called energy efficiency gap, i.e., the notion that there is under-investment in energy efficiency. Engineering studies often identify energy efficiency investments in the residential sector that should easily cover their own costs through the energy saved alone and come with additional or co-benefits from reductions in greenhouse gas emissions and other pollutants. The energy efficiency *gap* arises because these investment opportunities are often not realized. This creates a puzzle as to why society or individuals do not seize on these profitable investments? A number of studies have even attempted to quantify it empirically (see, for instance, McKinsey and Co 2009, and Dietz 2010, for the US economy and Ó Broin et al. 2015a, for the Swedish residential sector). Approaching the question in another way, studies have also evaluated specific energy efficiency investment programs including Metcalf and Hassett’s (1999) analysis of returns to insulation improvements, Levinson and Niemann (2004), Jacobsen and Kotchen (2013), and Kotchen’s (2017) analyses of the energy efficiency savings associated with building-code standards. A wide variety of potential explanations for the energy efficiency gap have been put forward. These include market failures such as imperfect information, principal-agent problems, split incentives, and capital market failures as well as behavioral explanations such as inattentiveness and reference-point phenomena (see, among others, Jaffe and Stavins 1994; Sorrell and O’Malley 2004; Nawrotzki 2012; Allcott and Greenstone 2012; Tsvetanov and Segerson 2013; Gillingham and Palmer 2014; Gerarden et al. 2017). Many dwellings are rented and split incentives are created since it is usually the owner of a building who takes decisions concerning insulation, maintenance, and other investments to reduce energy demand. If this is not the same person who pays energy bills, we have a reason for inefficiency that may be enhanced by the other factors mentioned. These explanations have made some economists question the existence of any significant savings from energy efficiency investments and led them to propose that the real-world returns on energy efficiency investments are lower than engineering models estimate. Fowlie et al. (2018) conduct a large-scale field experiment with approximately 30,000 households within the largest US residential energy efficiency program—the Weatherization Assistance Program. Their study finds that many investments were in fact not cost-efficient despite the promise from engineering models. However, many efficiency investments in buildings and their energy systems around the world should still be socially desirable and, for these, carbon pricing or other strong policies will be needed to internalize climate benefits and make them cost-efficient for both homeowners and society.

Micro studies have a big advantage in that a rich set of technical and behavioral variables can be included, preferably set in the context of a proper experimental setup. By contrast, macro studies of energy demand in buildings use aggregate country or state-level data. They have, in a way, the opposite advantages and disadvantages as compared to micro studies. Macro studies are relatively scarce compared to micro studies. However, some key studies have been conducted using US state-level data. Salari and Javid (2016) estimate the determinants of residential gas and electricity demand and compare static and dynamic estimates. Alberini and Filippini (2011) employ dynamic panel data estimators on US residential electricity demand and find a negative relationship between price and electricity consumption, while Filippini and Hunt (2012) employ a similar dataset and examine the underlying efficiency of US states.

Although there are some studies that investigate determinants of residential energy demand for individual European countries (Mata et al. 2021), only a handful of macro studies use cross-country data in the EU. Their focus has been primarily on identifying the effect from different categories of policy on energy efficiency (Filippini et al. 2014; Ó Broin et al. 2015b; Thonipara et al. 2019). This is difficult to do empirically at the macro level and inevitably requires major simplifications. Filippini et al. (2014) include dummy variables that indicate whether a country has implemented a certain number of policies that fall under three different policy categories, namely regulation standards, financial incentives, and information measures. Ó Broin et al. (2015a) use the same policy categories, but instead of using the number of policies in place, they weigh them by their expected effectiveness. Thonipara et al. (2019) highlight the issues of rough measures of policy effects as these and (as a complement to their quantitative demand analysis) choose a qualitative approach to study policy in a smaller set of countries. While most of these studies include price as a control variable in their demand equations, they seldom focus on the policy relevance of energy pricing.

These EU studies employ exclusively static model specifications. Bertoldi and Mosconi (2020) on the other hand use dynamic models to identify the effect of policy intensity in the residential sector and several other sectors. They include price as a control variable. As do those who individually study gas and electricity demand in the EU residential sector (Asche et al. 2008; Krishnamurthy and Kriström 2015). To the best of our knowledge, only two articles that cover EU countries study energy demand using dynamic estimation methods in an effort to estimate price elasticities. One examines ten OECD countries over the period 1970 to 1993 (Haas and Schipper 1998). The other explores the determinants of energy efficiency levels in merely four European countries using annual data from 1970 to 2005 (Ó Broin et al. 2015c). Both these studies find a negative relationship between price and energy use. We contribute to this strand of literature by employing dynamic panel data estimators on residential energy demand for all EU countries, estimating reliable and up-to-date estimates of short and long-run price and income elasticities for the EU.

Our study also stands out from the existing literature in three other important ways: (i) we incorporate prices for district heating in our price variable, which has previously only been done by Ó Broin et al. (2015b, c) and (ii) while the vast majority of previous studies analyze the determinants of different measurements of energy efficiency (most common are energy per capita or per household), we focus on demand at the aggregate level. This is because it is absolute energy use that is most relevant for the climate and because the number of persons per house is also partly endogenous as people typically choose more space with increasing economic affluence. Lastly, (iii) we provide estimates for total residential energy demand as well as for the end-use space heating and compare the results.

## Methodology

In this section, we describe our data and illustrate them by giving an overview of the variation in energy use between countries and over time. We then discuss the empirical strategy as well as the choice of estimators.

### Energy demand and efficiency data

We have constructed a dataset of annual residential energy consumption spanning from 1990 to 2018 for the EU-27 countries and the UK. The bulk of our data is secondary macro data from several distinct databases (Odyssee, Enerdata, Mure, AMECO) that we have combined and interpolated for missing data. All in all, our dataset includes energy use in the residential sector (both total and the space heating component), energy prices per energy carrier, personal income, outdoor temperature as measured by heating degree days, total floor area in the residential sector, and policy variables. We have constructed the energy consumption variable by aggregating time series of energy use of coal, district heating, electricity, gas, and oil for each country. Prices were similarly computed by weighting prices for all five energy carriers. Due to some missing data points for some of our variables, our panel is unbalanced. A detailed description of the construction of our dataset is available in Supplementary Note 1.

**Fig. 1:**
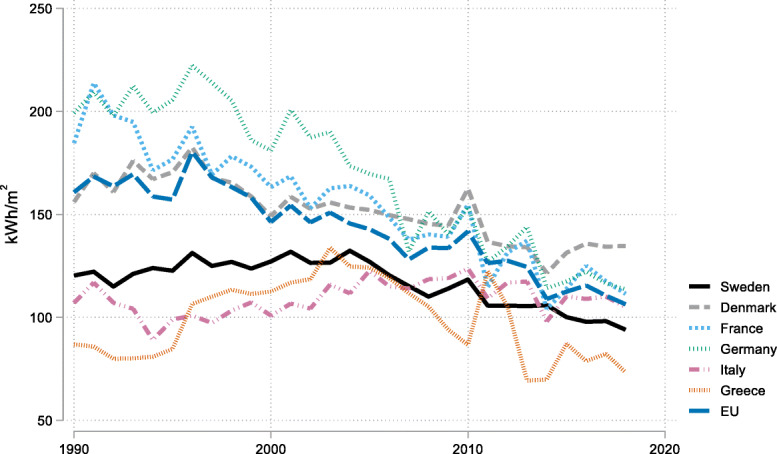
Average final energy demand for space heating (kWh/m^2^). NB residents in northern countries such as Denmark and Germany tend to consume more energy for heating per unit of floor area than residents in countries further south, such as Italy and Greece. Sweden has energy demand levels around the EU average despite having one of the coldest climates in the EU

The demand for heating energy has been fairly constant over time the last three decades in the EU. This pattern reflects a trend in increasing floor area per dwelling but at the same time a general decrease in energy requirements per unit of floor area (kWh/m^2^) although there are also big disparities between countries with different climates (Fig. 1).

### Empirical strategy

We take as our starting point the model of a generic energy demand function at the household level with climate, income, prices, demographic, and policy variables:

1 $$ {E}_{i,t}^{\ast }=f\left({P}_{i,t},{Y}_{i,t},{W}_{i,t},{x}_{i,t}^{\prime}\right) $$

${E}_{i,t}^{\ast }$ is the desired, or equilibrium, energy demand at time *t* in country *i*. *P*_*i*, *t*_ is the price of energy and *Y*_*i*, *t*_ is the net disposable income. Actual demand adjusts slowly to this equilibrium (see Eq. (2)). *W*_*i*, *t*_ is the climate (represented by the annual number of heating degree days). The energy demand variable can be either total residential energy demand or energy demand for space heating. Our main focus is on “total energy” which also includes electricity from computers, lamps, and cooking stoves etc. One reason for this is that electricity used in these appliances will normally add to warming the house or apartment (in summer extra heat from appliances may however be unwanted and be ventilated away). If there were a trend of increasing or decreasing electricity use for end uses other than heating, it could thus cause a compensating change in heating demand. The vector ${x}_{i,t}^{\prime }$ contains intermediate and exogenous variables that may influence demand (such as floor area, number of dwellings, and population) but are largely determined by socio-demographic and policy variables, such as income and energy price. These are the explanatory variables used in seminal papers on energy demand in buildings from Haas and Schipper (1998) and Filippini et al. (2014). It can be a delicate choice of whether or not to include intermediate variables. Inclusion may be useful since they add explanatory power and reduce the risk of omitted variable bias; however, since these variables also depend on for example income, this implies that the estimated income elasticity should be interpreted differently. Inevitably, there are many variables that are relevant to some degree that cannot be included since we do not have aggregate national data for them. If there are changes in such unobserved variables, then a time trend might be included as a proxy and it is therefore an alternative to include it as a so-called Underlying Energy Demand Trend (UEDT) as defined by Hunt et al. (2003a, b). It represents technical progress and other unobserved factors such as institutional and regulatory ones that explain energy consumption. Including the UEDT in our model allows us to control for “exogenous” technical progress that is determined by factors other than the price and thus only capture the “endogenous” technical progress in the price effect. We model the UEDT in two ways. First, by employing time dummies to allow for non-linearities (as advocated by Hunt et al. 2003a, b, and implemented in a panel data setting by Griffin and Schulman 2005 and Adeyemi and Hunt 2007) and, second, as a linear trend (since the dummy approach has been linked to estimation problems in similar settings by Kumbhakar and Lovell 2003 and Filippini and Hunt 2011). The vector ${x}_{i,t}^{\prime }$ may however still include some policy effects that are not captured by the UEDT. As an attempt to control for these, we follow a dummy variable approach as suggested by Filippini et al. (2014) where we create dummy variables of subcategories of policies to model energy performance standards, financial measures, and informational measures. These policy measures are all sourced from the MURE Policy Database (Enerdata 2021b), which covers hundreds of measures in Europe such as the French “Opération programmée d’amélioration de l’habitat” (OPAH) that offers engineering and financial assistance to rehabilitate buildings. This approach involves creating separate dummy variables for cases of 1–2 or >3 policies in any of the three policy categories that are in force at the time, i.e., a potential six different dummy variables. For more details on these variables, see Supplementary Note 2.

The long lifespan of buildings necessitates a serious focus on the dynamics of demand adaptation. Households cannot significantly change their consumption levels in direct response to shocks in price, income, or other variables. Adapting habits as well as equipment and building features takes time and, in the short run, the capital stock is almost fixed^1^. It is politically imperative to understand that people may thus feel “locked in” to their heating system and are very vulnerable to price increases^2^. For these reasons, we model demand responses through partial adjustment models.

The *desired* energy demand is the hypothetical demand from consumers if the capital stock were malleable. *Actual* demand represents a partial adjustment from historical levels to the desired one. The simplest linear representation of this is (2):

2 $$ {E}_{i,t}-{E}_{i,t-1}=\theta \left({E}_{i,t}^{\ast }-{E}_{i,t-1}\right) $$

where *E*_*i*, *t* − 1_ is the lag of actual demand and *θ* ∈ [0, 1] is a coefficient reflecting the adjustment speed. Equation (2) thus means that the actual change in energy use from year *t* − 1 to year *t* will adapt by a fraction θ to the desired change. Inserting the expression for ${E}_{i,t}^{\ast }$ from a linearized version of (1) into (2) yields the partial adjustment model:

3 $$ {E}_{i,t}=\upalpha +\gamma {E}_{i,t-1}+{\beta}_1{P}_{i,t}+{\beta}_2{Y}_{i,t}+{\beta}_3{W}_{i,t}+{x}_{i,t}^{\prime}\boldsymbol{\beta} +{\epsilon}_{i,t}. $$

where the coefficient parameter *γ* = 1 − *θ*, *α* is the constant, *β*_*k*_ is the coefficient parameter of the *k*th regressor, and *ϵ*_*i*, *t*_ is the error term. In this study, we chose a log-linear form in which *β*_1_ and *β*_1_/(1 − *γ*) can be interpreted as the short- and long-run price elasticities respectively.

### Choice of estimation methods

To estimate a dynamic demand equation as in (3), model choice is central as estimated elasticities vary between methods (Espey and Espey 2004; Alberini and Filippini 2011; Salari and Javid 2016). We employ both random-effects (Generalized Least Squared; GLS) and fixed-effects (Least Square Dummy Variable; LSDV) estimators. While there is a risk of omitted variable bias when using the GLS estimator in this setting, we include it since it gives an upper bound by attributing all the variation observed to the included explanatory variables. However, we also employ fixed-effects models to control for all time-invariant characteristics between countries and study only the variations within countries. When performing a Hausman test, we indeed find that the unique errors (*ϵ*_*i*_) are correlated with the regressors, thus indicating that a fixed effects estimator is to be preferred. However, since *E*_*i*, *t*_ is a function of *ϵ*_*i*_ in Eq. (3), it follows that also *E*_*i*, *t* − 1_ is a function of *ϵ*_*i*_. Hence, the regressor *E*_*i*, *t* − 1_ is correlated with the error term, which makes the LSDV estimator biased and inconsistent for a finite T (see Nickell 1981). Despite this possible bias in estimates, the LSDV estimator (as well as the GLS estimator) is commonly applied in the existing energy demand literature and estimated results are thus of reference value. The bias does not vanish as the number of entities (N) increases, but the LSDV estimator becomes consistent as T grows (the endogeneity bias → 0 as T → ∞). In the present study, T = 29 (and N = 28). Monte Carlo experiments have been performed with N set to 20 while alternating the time dimension (Judson and Owen 1999). It is found that, for unbalanced panels, when T = 30, the LSDV estimator performs just as well than most viable alternatives. Thus, since the endogeneity problem might not be severe, and for the reference value it serves, we estimate LSDV results for energy demand in this study. Robust standard errors adjusted for clustering are used for the LSDV estimations to account for possible heteroscedasticity.

Since the endogeneity issue of a lagged dependent variable was first discussed in the econometric literature, a number of consistent instrumental variable and Generalized Method of Moments (GMM) estimators have been suggested as alternatives to the LSDV estimator. It was suggested early on to transform the equation in first differences and use the second lags of the dependent variable (either in levels or differenced) as instruments for the one-time lagged dependent variable (Anderson and Hsiao 1982). Later, it was argued that it is a more efficient alternative to use a greater number of internal instruments (Arellano and Bond 1991). This approach was further developed by restricting initial conditions, and a “system” GMM estimator was shown to be more efficient and stable (Blundell and Bond 1998). This development also lowers the risk of any issues with small-sample bias (Hayakawa 2007). We employ this estimator and denote it BB-GMM. All GLS, LSDV, and BB-GMM regressions presented in this paper are population-weighted.

Another possible endogeneity issue, in addition to the lagged energy use, may come from the price variable. Following basic demand-supply theory, the price variable should be determined simultaneously by demand and supply functions. Partial analysis of the demand function alone might be biased depending on whether prices are exogenous. In an effort to mitigate the risk of such endogeneity bias, we use all feasible lags of the price as instruments in our BB-GMM estimates (similarly to Alberini and Filippini 2011). We do however not expect this endogeneity to be a major issue in the context of this study (see Flood et al. 2010, for a somewhat similar analysis in the context of transport fuel demand). Firstly, there are multiple other usages besides residential use for the concerned energy carriers. This limits any demand effect since, for instance, a drop in demand in the residential sector and the downward pressure on prices that follows could possibly yield a counteracting increase in demand for energy from the other sectors. Secondly, many of the energy carriers are commodities that are traded on a global market, hence making the energy consumption for space heating in the six countries included in this study a negligible share of total global demand. It is unlikely that the relatively small changes in demand for these countries that can be observed over time influence, e.g., global oil prices in a significant way. Lastly, a significant share of the energy price is determined by local policies. At the aggregate level, the potential for price to be endogenous with consumption is mitigated by the many different pricing levels and schemes at different locations (Shin 1985). For this reason, previous studies of electricity demand have considered average prices as exogenous (Bernstein and Griffin 2006; Paul et al. 2009).

## Results

In this section, we discuss the econometric estimation of price, income, and other effects. We start with the main results and elasticities in Section 4.1. In Section 4.2, we focus on country-specific effects and Section 4.3 contains some comments and discussion of results.

### The effect of energy prices and income as well as climate

**Fig. 2:**
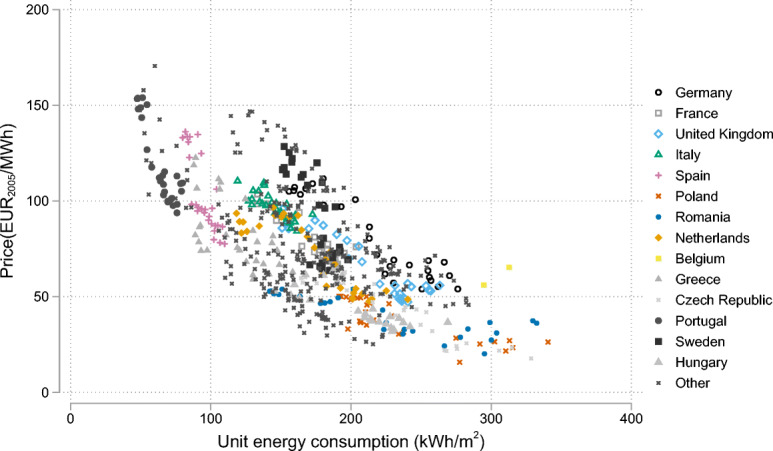
Residential energy—the demand curve

Figure 2 shows a negative relationship in our data between energy consumption per unit of area and price in countries covered (the 14 most populous are labeled) from 1990 to 2018 (the equivalent plot for space heating is available in Supplementary Figure S1). Our regression analysis confirms this negative relationship and examines additional variables such as income, climate, and other conditions.

Table 1 presents estimates from the GLS, LSDV, and BB-GMM estimators of Eq. (3) for both total residential energy demand and energy demand for space heating. In addition to the usual demand determinants of price and income, the ambient outdoor temperature, which is our proxy for climate, is of course a central variable in this setting and hence we include it as a control variable. We see in Table 1 that the climate parameters are positive (colder climate yields higher energy consumption) and have a high impact, and this effect is found to be slightly larger for space heating than total demand. As can be noted in Table 1, the difference in estimates between our two energy measures is small. One difference is that the inertia as captured by the coefficient of the lag variable is greater for space heating, yielding higher long-run price elasticities—indicating that pricing policies are *especially effective* for heating.

**Table 1:** Regression estimates of residential energy demand. All parameter estimates displayed can be interpreted as elasticities

	Total residential energy demand			Space heating demand		
	[1] GLS	[2] LSDV	[3] BB-GMM	[4] GLS	[5] LSDV	[6] BB-GMM
Energy lag	0.914*** (0.029)	0.767*** (0.039)	0.909*** (0.030)	0.922*** (0.019)	0.736*** (0.036)	0.919*** (0.020)
Price	−0.061** (0.027)	−0.066*** (0.017)	−0.064** (0.028)	−0.064*** (0.022)	−0.097*** (0.018)	−0.067*** (0.023)
Income	0.076*** (0.026)	0.056** (0.025)	0.080*** (0.027)	0.077*** (0.016)	0.084***(0.019)	0.080*** (0.018)
Climate	0.059** (0.022)	0.312*** (0.066)	0.062** (0.023)	0.121*** (0.041)	0.558***(0.087)	0.125*** (0.044)
Constant	−0.206 (0.146)	−1.525*** (0.505)	−0.222 (0.156)	−0.797** (0.316)	−3.527*** (0.669)	−0.822** (0.335)
Long-run price elasticity	−0.707***	−0.283***	−0.702***	−0.828***	−0.368***	−0.825***
Long-run income elasticity	0.885***	0.240**	0.886***	0.992***	0.320***	0.995***
Sargan *p*-value			0.981			0.971
AR1 *p*-value			0.005			0.003
AR2 *p*-value			0.147			0.212

Robust standard errors in parentheses

*p<0.10; **p<0.05; ***p<0.01

We further note in Table 1 that the GLS estimator provides larger elasticity estimates than the LSDV estimator since it utilizes all the variation in the data while the LSDV estimator is restricted to the within-country variation (see columns [1–2] and [4–5]). So is the BB-GMM estimator that we introduce in a third step that additionally accounts for the potential endogeneity in the lagged dependent variable as well as the price variable. While doing this, we again note an increase (in absolute value) of the long-run price and income elasticities to a similar level as the GLS estimates (column [3] and [6]). Of these three estimators, we prefer the BB-GMM estimator but include the others for comparison.

The income elasticities provided in Table 1 can be viewed as the total income effect emanating from two main channels: the demand for larger dwellings and demand for greater comfort or indoor temperature. To distinguish between these, we can introduce the intermediate variable floor area. Table 2 shows the results for total residential demand. The advantage of including such intermediate variables is a richer explanation of demand but this comes with implications since floor area, in turn, depends strongly on income. The inclusion of this variable reduces the parameter estimate for the lagged endogenous variable. Thus, the long-run income elasticity drops dramatically from 0.89 from our preferred estimator in Table 1 (column [3]) to 0.31 (column [1] in Table 2), but we must interpret this latter value as a partial elasticity: the effect of income—in addition to the rise in floor area. The price elasticity is also somewhat affected: The short-run elasticity increases from 0.06 to 0.12 while the long-run value falls from 0.70 to 0.57 due to the lower estimate for the lagged endogenous parameter.

**Table 2:** BB-GMM regression results of extended demand function specifications of total residential energy demand. Including floor area, non-linear and linear UEDT, and policy dummies as regressors stepwise. All parameter estimates displayed can be interpreted as elasticities

	[1] Including floor area	[2] Including non-linear UEDT	[3] Including linear UEDT	[4] Including policy dummies
Energy lag	0.784*** (0.039)	0.776*** (0.046)	0.872*** (0.017)	0.770*** (0.045)
Price	−0.124*** (0.023)	−0.105*** (0.019)	−0.064*** (0.012)	−0.110*** (0.022)
Income	0.067*** (0.020)	0.051*** (0.017)	0.033*** (0.011)	0.054*** (0.018)
Climate	0.135*** (0.032)	0.147*** (0.039)	0.069*** (0.014)	0.151*** (0.041)
Floor area	0.162*** (0.037)	0.188*** (0.050)	0.102*** (0.021)	0.192*** (0.047)
Constant	−0.938*** (0.262)	2.319** (1.110)	−0.558*** (0.150)	1.536 (1.161)
Long-run price elasticity	−0.574***	−0.469***	−0.498***	−0.478***
Long-run income elasticity	0.308***	0.229***	0.260***	0.232***
UEDT	No	Dummies	Linear	Linear
Policy dummies	No	No	No	Yes
Sargan *p*-value	0.620	0.699	0.438	0.617
AR1 *p*-value	0.004	0.004	0.001	0.004
AR2 *p*-value	0.193	0.198	0.690	0.197

Robust standard errors in parentheses

*p<0.10; **p<0.05; ***p<0.01

Next we control for exogenous technical progress and other exogenous factors by including the UEDT both non-linearly using dummies (Table 2, column [2]) and linearly (column [3]) in the model for total demand. In line with our expectations, the introduction of the UEDT reduces the elasticity estimates somewhat further. In our final specification (column [4]), in an effort to control for policy measures not captured by the UEDT, we also introduce the policy dummies for building policies described in Section 3.2. While the effect of the dummies is jointly statistically significant at the 10% level, they are not individually statistically significant. As discussed by Thonipara et al. (2019), this may just be too rough of a measure of policies to draw conclusions from them individually. Collectively, they do still contribute with explanatory power however and thus we feel that this model specification merits particular attention. With this preferred specification, we find short- and long-run price elasticities of −0.11 and −0.48 respectively. Thus, in the long run, households respond (ceteris paribus) to a 10% increase in price with approximately a 5% reduction of energy consumption.

#### Validity of instruments and robustness checks

We test the validity of the instruments used in the BB-GMM estimator using the Sargan test of over-identifying restrictions and present the resulting *p*-values in the regression tables. The high *p*-values found (see Table 1 and 2) indicate that the instruments are valid. We further employ the autocorrelation test suggested by Arellano and Bond (1991) and Blundell and Bond (1998) to test for the existence of first (AR1)- and second (AR2)-order serial correlation among the error terms. The *p*-values from the test results, as presented in Tables 1 and 2, indicate that there is no significant second-order serial correlation, which is vital for the validity of the instruments.

We further replicate the estimations presented in Table 2 also for space heating demand. The results are available as Supplementary Material in Table S1. Again, we can observe somewhat larger price elasticities in the long run for this measure of demand compared to total residential demand. We also replicate the estimation results of our preferred model specification using the alternative estimators and present the results in Table S2. We can note from these results that the results of the LSDV estimator and the BB-GMM estimator are rather similar, indicating that the endogeneity issues that are accounted for in the BB-GMM estimations may not be severe. As a further test of the sensitivity to estimator choice, we here also employ the bias-corrected estimator (LSDVC), which has been shown to perform well with small samples (Judson and Owen 1999) and has previously been applied to energy demand estimations at the macro level (Alberini and Filippini 2011)^3^. When applying this estimator, we find a somewhat higher level of inertia than what we found using the BB-GMM estimator and larger long-run price elasticities (Table S2, column [3]). However, the estimates are roughly the same and we can conclude that our results are robust regardless of whether we account for potential endogeneity issues in the lagged dependent variable by using an instrumental variable approach (BB-GMM) or a bias correction approach (LCDVC).

As a further test of heterogeneity, we split the sample into two regions: Western and Eastern Europe. From the results in Table S3, we can note that the price effect is not statistically significant for the Eastern European countries but only in Western Europe. A possible interpretation might be that energy prices do not play such a role for residential energy demand in these formerly planned economies.

### Effects of country-specific effects and policy measures

As mentioned, energy efficiency depends on many variables such as building standards and we do our best to control explicitly for the impact of these measures (using the approach from Filippini et al. 2014). In spite of this, some additional effects of unobserved policy activity are likely to be captured by the country dummies in our fixed-effects regressions together with some “underlying” energy efficiency of an individual country. Thus, we can glean some aggregate information by looking at country fixed-effects coefficients in our regressions to identify the sum of numerous excluded and often subtle differences between both social norms of behavior and the accumulated effect of architecture and building technology policies.

**Fig. 3:**
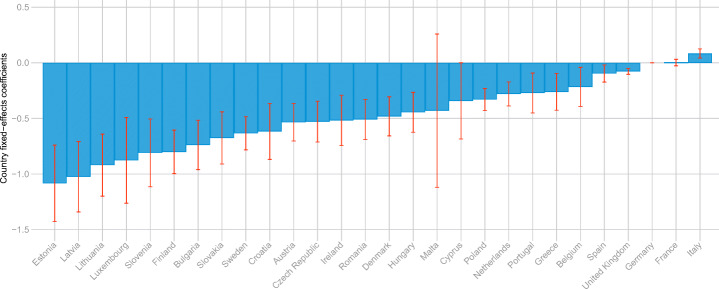
Country fixed effects. The figure displays the country fixed-effects coefficients from our full model specification. Germany is the reference and therefore has a value of 0

Warm countries in the south of Europe generally consume (somewhat) less energy for heating per floor area unit. The climatic reasons for this are obvious. However, as is shown in Figure 1, Sweden uses no more than the EU average in spite of a cold climate. It seems that efficiency or high investments in building standards compensate for the cold climate. Figure 3 explores country fixed-effects coefficients and shows that Sweden has a fairly large negative fixed effect. In fact, many of the countries with the largest negative fixed effects are cold countries in Scandinavia, the Baltic, or the Alps regions. These fixed effects may be a reflection of an adaptation to colder climate and possibly other factors such as policy, demographics, or income not already captured by our variables.

### Discussion

Standard economic theory and common sense suggest that energy demand increases with income and decreases with energy price. As we discussed above with reference to engineering and microdata analyses, there are a number of factors that mediate the simple relationship in the demand for heating or energy in residential (and commercial) buildings. Larger buildings and the desire for more comfort will increase the use of energy. On the other hand, there is clearly also a cost associated with well-designed, weatherized high-efficiency housing that provides a comfortable indoor climate without large amounts of external heating energy. So increased income could lead, under the right conditions, to lower energy demand. In addition to this, there are numerous market and policy imperfections or failures. In apartment houses for instance, a very large fraction of the heat that escapes one apartment will go to neighboring flats. There are split incentives between those who build, own, and occupy. There are technical complications of various kinds, market failures such as asymmetric information, limited competition, technical externalities, and behavioral anomalies and cognitive limitations and biases that prevent us as consumers from taking fully “rational” (long-run cost-minimizing) decisions. It is not always immediately apparent to the user what the costs of his or her actions are. These factors might reduce the price elasticity but are unlikely to eliminate it completely.

Our estimated price elasticities of −0.1 in the short run and −0.5 in the long run can be compared with the few estimates we have from the previous comparable literature. For the US, Salari and Javid (2016) find short- and long-run price elasticities of −0.56 and −1.09 respectively for residential gas demand and −0.07 and −0.31 for electricity demand. The latter can further be compared with the results provided by Alberini and Filippini (2011) that find price elasticities for the US residential electricity demand of −0.15 in the short run and −0.73 in the long run. For the EU, the only comparable estimate that has previously been estimated is the one by Ó Broin et al. (2015c) that find an average long-run elasticity of −0.25 for residential heating demand for France, Italy, Sweden, and the UK^4^. Other pooled estimates are from static models and imply price elasticities within the range of −1.22 to −0.14 (Ó Broin et al. 2015b; Thonipara et al. 2019).

The price sensitivity of energy demand we find is intermediate to high. This is important information for policy design. Many significant policies like carbon or energy taxes or green certificates act by modifying the price of energy that consumers pay. The elasticities we estimate are averages for all buildings. Had all buildings been owner-occupied, the price elasticity would presumably have been higher (Gillingham et al. 2012; Broberg and Egüez 2018). Split incentives and energy poverty also imply that there is strong resistance to using energy prices too aggressively since low-income tenants could be hurt. This in turn implies that complementary policies are necessary that incentivize or pressure building owners to ensure their efficiency. This can mean that energy prices are complemented by subsidies that for instance lower the cost of energy-saving investments. In the case of carbon taxes, the dividends can be redistributed or targeted in socially sensitive ways.

In the light of the gravity of the climate challenge, many observers and policymakers are interested in energy use primarily because they are interested in climate policy. In addition to energy efficiency, the greenhouse gas emissions of buildings in a country are also affected by specific investments in fuel switching and in infrastructure. This can be illustrated by the district heating sector in Sweden which has grown from 10 to over 50% coverage since 1970. Simultaneously, district heat systems have switched from being mainly based on oil to using primarily low emission sources such as waste heat, biomass, and heat pumps.

## Conclusions, policy implications, and further research needs

This paper studies the determinants of residential energy demand and focuses primarily on the economic determinants of energy demand. By employing dynamic panel data estimators, while controlling for the effect of other policies and country-specific effects, we analyze a uniquely compiled macro-level dataset of EU residential buildings to provide estimates of price (and other) elasticities that should be useful in future modeling and policymaking. We are the first to simultaneously provide EU-wide (long and short run) price and income elasticities for both total energy demand and space heating energy demand using the same dataset.

We find that income is a clear determinant of total and space heating demand for European households. Climate policy often assumes demand is given and will be reduced if policies are implemented. There is however a clear risk that demand could increase with rising income and thus some price increase would be required just to keep demand constant. It is for this reason important to have good and consistent estimates of the elasticities. Our results show that income increases demand in two ways—directly and indirectly through its effect on intermediate variables such as floor area. The direct elasticity may be as low as 0.2 while the total elasticity is of the order of 0.9.

When it comes to the price elasticity, we find important dynamic effects. Short-run effects of increased price might be −0.1 or lower but in the long run, price elasticities may well be of the order of −0.5. This implies that we have a chance of affecting residential energy consumption using price instruments. The price and income elasticities found suggest that even when policymakers rely on regulations and other policies to reduce demand, they also need to remember the risk for rebound effects (and simply the effects of increased affluence) so that regulations need at the very least to be backed up or supported by increasing prices. Based on the elasticities presented in Table 2, a tax that raises the effective price of energy by 10% would reduce demand by a modest but still considerable amount of 5%. This translates to a total reduction in energy demand of over 250 TWh in the residential sector in the countries studied. In energy systems that are based on fossil fuels, such price increases could be achieved by imposing a carbon tax (or cap-and-trade system). In such a scenario, the reduction in energy consumption would in all likelihood achieve additional reductions in carbon dioxide emissions through fuel switching (Ó Broin et al. 2019b). However, as the dynamic econometric results indicate, pricing policies are especially effective in the long run: Replacing heating systems and other capital stock in the residential sector comes with certain costs and other constraints and households are thus to some degree locked in with their current system. As the elasticities presented in Table 2 show, the short-run effect would only be a fifth of the long-run response. It is thus important to use effective instruments such as prices—but also to understand the distributional challenges posed, particularly in the short run.

On the one hand, the high inertia and slow response rate mean that policies must be introduced soon to meet any given target. On the other hand, this discrepancy over the short and long run means that the politicians backing the decisions must take into account the difficulties faced by those bearing higher costs now while the benefits (reduced bills and smaller environmental impact) come only in the long run. To overcome some of the political challenges and increase feasibility, it may help not only to increase prices but also—at the same time—lower costs of retrofitting energy systems and houses (through subsidies or tax breaks), especially for low-income households. This is one of the motivations for the many programs of support for various measures that can be found in most countries.

In order to explore better the special demands of a fossil-free buildings sector, future work will need to be focused more on the determinants of energy switching and on the demand for individual energy technologies and carriers, e.g., district heating, heat pumps, or natural gas demand in the EU. The methods used in this study could be applied to the residential sectors of other global regions provided sufficient data are available. Other possibilities would be to extend the study to include commercial buildings although one needs to be cognizant of the different energy demand profiles of hospitals, restaurants, hotels, and offices where micro studies may sometimes be a good complement. In the future, a warming climate and more stringent building standards will likely reduce the need for heating in Europe but perhaps increase the demands for air conditioning which may have quite different determinants. To conclude, we believe that a combination of micro and macro-level studies will be needed to understand the new energy demand patterns that emerge in the future.

## Supplementary Information

ESM 1 (PDF 848 kb)

## Data Availability

Available upon reasonable request.

## Footnotes

1 The capital stock in this context is mainly the building although also infrastructure, heating systems and even knowledge is sometimes included in the concept. Adaptation mechanisms can include both changed habits and actual modifications to building or equipment.

2 These features are often reinforced by the institutional rules and conditions such as the common existence of tenant-landlord relations that imply split incentives.

3 We incorporate the corrections to accommodate unbalanced panels as suggested by Bruno (2005) for this estimator.

4 Haas and Schipper (1998) find negative price elasticities in the residential sector in Japan, Sweden, and Australia ranging from −0.20 to −0.05. From the regression tables presented by Bertoldi and Mosconi (2020), a small negative relationship between energy consumption and price can be observed. Davis (2021) further finds that energy prices are vital for electrification in the USA.
