# Supplementary material for: Saving energy in residential buildings: the role of energy pricing
Source: Clim Change. 2021 Jul 21;167(1-2):18. doi: 10.1007/s10584-021-03164-3 (PMC8294229; doi:10.1007/s10584-021-03164-3)
Supplement: Supplementary file 1 — (PDF 848 kb) [file 10584_2021_3164_MOESM1_ESM.pdf]

Supplementary Material

Supplementary Note 1

Data collection and harmonization
The spatial focus of this work is the residential sectors of the 27 individual countries of the European Union and the United Kingdom. The temporal focus is each year from 1990 to 2018.

Table S4 lists the variables used in the regression models in this work and how they have been arrived at. Data for both total energy consumption in the residential sector and energy consumption for space heating, are the sum of the respective energy consumption of coal, oil, natural gas, electricity and district heating for each. These aggregate consumption variables are different from standard data available for total energy consumption and energy consumption for space heating in that they do not include the use of biomass. Although data on biomass consumption for both total energy demand and space heating demand is available from the data sources used, corresponding data on prices for biomass are not. Data for the prices for the other five energy carriers is available meaning that the biomass component of total demand is removed to make the price and demand statistics correspond. Prices of biomass are not traditionally collected in databases due to its trade mostly being a cottage industry. The total energy consumption variable combines energy consumption for space heating, electrical appliances (including HVAC), water heating, cooking and lighting. Units of terawatt-hours are used in this work.

Time series of total energy consumption and space heating consumption for the five energy carriers (coal, oil, gas, district heating, and electricity) used was obtained from the Odyssee Database (Enerdata 2021a). The Odyssee database harmonizes time series of data categories for energy end-uses and related drivers from the national energy agencies of individual EU countries. While both the IEA and Eurostat collect and harmonize national-level data on usage of energy carriers from the same energy agencies, they do not do so for end-use level data e.g. residential space heating energy demand.

For our price variables, we use the weighted average price (WAP) for total energy consumption and space heating energy consumption, respectively. We constructed the WAP by weighing the time series of prices for each of the five energy carriers in each country, by the corresponding time series of their consumption in the same country. Time series of prices for the residential sector of coal, oil, gas, and electricity normalized to year 2005 prices in euro were obtained from the Global Energy and CO_2 database (Enerdata 2020). Enerdata, who compile this database, take price data from the IEA and combine them with currency exchange rates and deflators from the World Bank to create a time series of energy prices normalized to euro 2005. The time series of district heating prices were extracted from Werner (2016) and was also in parts provided directly from the author. These district heating prices were without taxes, and thus data on VAT, excise taxes and carbon taxes were obtained from national sources for each country where district heating was used to estimate the prices paid by consumers. For the purposes of this work, they were then normalized to year 2005 prices using the same deflator and exchange rates used by Enerdata. At the time of writing Enerdata have a new database of district heating prices to 2019 which may be used in further work.

The AMECO database by the European Commission (AMECO 2021) provided nominal income data for the net disposable income: households and NPISH (non-profit institutions serving households) category. For the purposes of this work, income is also normalized to euro 2005 prices using the same deflator and exchange rates as Enerdata use to create prices in euro 2005 prices.

We employ a proxy variable for the climate by the use of heating degree days (HDD) from Eurostat (2021) The calculation of HDD relies on the base temperature, which is defined as the lowest daily mean air temperature not leading to indoor heating (T_m). Eurostat set the base temperature to 15°C. If $T_m \leq 15°\mathrm{C}$ then $\mathrm{HDD} = \sum_j (18°\mathrm{C} - T_m^j)$ else $\mathrm{HDD} = 0$. T_m^j is the mean air temperature of day j. The data is collected on a daily basis and is added up to represent the sum of heating degree days over one year.

The floor area variable is constructed as the product of average floor area per dwelling and the number of permanently occupied dwellings. Both these variables along with population data were obtained from the Odyssee Database (Enerdata 2021a).

Data construction

For some data categories for some years data is unavailable. As a result of this, the panel is unbalanced in the sense that the time series for some countries do not cover the entire time period of year 1990 to 2018. For 28 countries and 29 years, a balanced panel would contain 812 observations. Due to missing data the actual number of data points is 674 (83% of potential balanced panel). Of the 674 data points, 229 are constructed in some part, i.e. one of the variables used is imputed uniquely for the purposes of this work. Thus, there is only a complete variable set for each country and year for 55% of the 812 points.

Figure S2 shows the countries and years where there is no missing data (in black), where there is at least one variable in a particular year for which imputation has been employed (grey) and where there are missing values in at least one variable (white). The variables covered in the table are those representing total energy consumption, price, income, climate, and floor area.

In the case of Austria, for example, the AMECO database does not have data for the income category used for years 1990 to 1994. For these years, data for income is imputed by extrapolating the year 1995 value from AMECO back to 1990 using the rate of change of GDP in Austria during this period. Thus, for the years 1990 to 1994 Austria is coloured grey due to one variable (income) being an extrapolation. We can also note, for example, that there is no data available on total energy demand in the Odyssee Database for Luxembourg for the period 1990 to 1999. Thus, regardless of the availability of other data categories, Luxembourg is excluded from the panel pre-2000.

Where energy prices are imputed, they are typically constructed relative to the time series of the prices of the dominant energy carrier used in the same country or by that of the same energy carrier in a neighbouring country. For the Czech Republic, for example, oil prices from 1990 to 1993 are imputed from those found in Slovakia. This is a reasonable assumption since both were united in Czechoslovakia during these years. Energy Consumption for Finland from year 1990 to 1994 is obtained from a private database for the period 1970–1996 constructed at the Precourt Energy Efficiency Center at Stanford University by the late Lee Schipper. As the price time series for district heating obtained from Werner (2016), are to 2013, prices for 2014 to 2018 are imputed for each country where district heating is used, based on the dominant energy carrier used for space heating in the country. Coal prices are missing for a significant share of countries post year 2000. In most cases this is inconsequential as the use of coal has become negligible in most countries. For example, there is no data for Austria on coal prices from 2006 to 2016. However, during that decade, coal demand had fallen to less than one TWh out of a total of over 50 TWh and hence coal was removed from the WAP for this period in this particular case. Our time series stops in 2018 due to there being a lag with the provision of data in most of the databases used.

For both Belgium and Malta, there is no data on floor area for the years filled in in white in Figure S2, e.g. 1990–2016 for Malta. Because of Brexit, there is no income data in the AMECO database for 2017-2018 for the UK. There is insufficient energy price data available for Bulgaria pre-2007 to construct a WAP. The cells coloured grey between 2014 and 2018 for all countries are due to the estimate of DH prices for the period, and also due to the estimation of Income for 2017 and 2018, with the AMECO category, Gross wages and salaries (D11; UWSH), due to the preferred data category, Net disposable income: households and NPISH, no longer being listed in AMECO for these years.

All imputations are described in detail in Table S5. Descriptive statistics of the complete dataset are presented in Table S6.

Supplementary Note 2: Dummy variable strategy for modelling policies

A typical econometric strategy to modelling the introduction of policy is to use dummy variables for the years in which they are in force. This is not straightforward however for our panel of 28 countries as there are more than 500 relevant policies in the MURE policy database (Enerdata 2021b) to account for over the period 1990–2018. Filippini et al. (2014) approach this by categorizing the policies e.g. if they are regulatory or informative focused, and to then have two possible dummy variables for each category depending on whether there are 1–2 or >3 policies in place. Following Ó Broin et al. (2015b), we divide the 500+ policies into three categories for this work, (i) building regulation, (ii) Grants and Subsidies, and (iii) Information focused policies. Filippini et al. (2014) found significant results using the threshold of two or less policies in place for their first dummy variable for each policy category and more than two policies in place for their second, and thus we use the same threshold.

Table S1. BB-GMM estimates with extended demand function specifications for "space heating".

	[1]<br>BB-GMM	[2]<br>BB-GMM	[3]<br>BB-GMM	[4]<br>BB-GMM
Energy lag	0.908***<br>(0.022)	0.907***<br>(0.024)	0.951***<br>(0.010)	0.905***<br>(0.023)
Price	-0.069***<br>(0.020)	-0.045**<br>(0.020)	-0.026*<br>(0.015)	-0.049*<br>(0.025)
Income	0.060***<br>(0.018)	0.039**<br>(0.015)	0.026**<br>(0.009)	0.045**<br>(0.020)
Climate	0.141***<br>(0.048)	0.152***<br>(0.054)	0.061**<br>(0.023)	0.165***<br>(0.055)
Floor area	0.041*<br>(0.024)	0.068**<br>(0.032)	0.028*<br>(0.014)	0.066*<br>(0.036)
Constant	-1.059**<br>(0.417)	2.656***<br>(0.924)	-0.533**<br>(0.237)	0.756<br>(1.112)
Long-run price elasticity	-0.747***	-0.481**	-0.520**	-0.523**
Long-run income elasticity	0.654***	0.419**	0.529***	0.475**
UEDT	No	Dummies	Linear	Linear
Policy dummies	No	No	No	Yes
Sargan p-value	0.948	0.956	0.361	0.928
AR1 p-value	0.004	0.004	0.001	0.004
AR2 p-value	0.258	0.268	0.991	0.266

Robust standard errors in parentheses
* p<0.10, ** p<0.05, *** p<0.01

Table S2. Preferred model specification of total residential demand with alternative estimators.

	[1] GLS	[2] LSDV	[3] LSDVC
Energy lag	0.783***	0.645***	0.826***
	(0.042)	(0.066)	(0.026)
Price	-0.103***	-0.166***	-0.104***
	(0.020)	(0.027)	(0.018)
Income	0.051***	0.041**	0.027***
	(0.017)	(0.017)	(0.009)
Climate	0.140***	0.496***	0.395***
	(0.040)	(0.058)	(0.033)
Floor area	0.180***	0.133*	0.011
	(0.045)	(0.069)	(0.047)
Constant	1.551	-5.994***	
	(1.117)	(2.043)	
Long-run price elasticity	-0.473***	-0.468***	-0.599***
Long-run income elasticity	0.234***	0.115***	0.158***
UEDT	Linear	Linear	Linear
Policy dummies	Yes	Yes	Yes

Robust standard errors in parentheses
* p<0.10, ** p<0.05, *** p<0.01

Table S3. Split-sample models for Western and Eastern Europe.

	[1] BB-GMM Western Europe	[2] BB-GMM Eastern Europe
Energy lag	0.859***	0.890***
	(0.028)	(0.023)
Price	-0.082***	-0.042
	(0.027)	(0.051)
Income	0.066**	0.031**
	(0.028)	(0.012)
Climate	0.071***	0.096**
	(0.022)	(0.038)
Floor area	0.080*	0.087**
	(0.043)	(0.037)
Constant	-0.397	-0.772
	(0.235)	(0.557)
Long-run price elasticity	-0.580***	-0.382
Long-run income elasticity	0.466**	0.282**
UEDT	Linear	Linear
Policy dummies	Yes	Yes
Sargan p-value	0.473	0.110
AR1 p-value	0.006	0.037
AR2 p-value	0.989	0.249

Robust standard errors in parentheses
* p<0.10, ** p<0.05, *** p<0.01

Table S4. Variables used in the regressions in this study and their composition.

Variable	Symbol	Unit	Composition	Source
Total energy consumption	E	TWh	Sum of total final energy consumption for Oil, Coal, Natural Gas, Heat and Electricity.	Enerdata (2021)
Space heating consumption	E	TWh	Sum of final energy consumption for space heating for Oil, Coal, Natural Gas, Heat and Electricity.	Enerdata (2021)
Electricity consumption	E	TWh	Final electricity consumption.	Enerdata (2021)
WAP total	P	€$_{2005}$/TWh	Prices for Oil, Coal, Natural Gas, Heat and Electricity in euro normalized to year 2005 and weighted by proportion of their consumption for total energy in residential sector to create a single price index for energy.	Enerdata (2020, 2021); Werner 2016)
WAP space heating	P	€$_{2005}$/TWh	Prices for Oil, Coal, Natural Gas, Heat and Electricity in euro normalized to year 2005 and weighted by proportion of their consumption for space heating to create a single price index for energy.	Enerdata (2020, 2021); Werner (2016)
Electricity price	P	€$_{2005}$/TWh	Electricity price for domestic users.	Enerdata (2021)
Income	Y	€$_{2005}$	Household and non-profit institutions serving households (NPISH) net disposable income normalized to Year 2005.	AMECO (2021)
Heating degree days	W	ºC	As described in Methods section.	Eurostat (2021)
Total floor area	-	km^2	Average floor area per dwelling multiplied by number of permanently occupied dwellings.	Enerdata (2021)

Table S6. Descriptive statistics of regression variables.

Variable	Symbol	Obs.	Mean	Std. Dev.	Min	Max
Total consumption	E	789	109.58	159.59	0.70	776.09
Space heating consumption	E	736	75.06	114.96	0.10	602.13
Electricity consumption	E	789	27.35	38.56	0.47	163.07
WAP total	P	706	74.37	28.80	15.80	170.54
WAP space heating	P	683	61.41	26.59	11.67	160.02
Electricity price	P	771	128.03	48.37	17.18	267.43
Income	Y	776	246.71	388.60	2.14	1643.53
Heating degree days	W	812	2909.59	1154.53	322.12	6413.20
Floor area	-	722	659.61	856.80	6.78	3568.59

Table S5. Detailed description on data imputation.

	**Data Treatment : Income** (Net disposable income: households and NPISH)	**Data Treatment : Energy Price**	**Data Treatment : Other**
Austria	1990 – 1994: Extrapolation from 1995 value using GDP time series.		
Bulgaria	1990 – 1996: Extrapolation from 1997 value using GDP time series		
Croatia	1990 – 2001: Extrapolation from 2002 value using GDP time series & 2013 – 2014: Extrapolation from 1995 value using earlier version of AMECO database.	Electricity price 2004: Interpolation between 2003 and 2005 price.	
Czech Rep.	1990 – 1994: Extrapolation from 1995 value using GDP time series	Price oil, gas, coal and dh 1990 – 1992: Extrapolation from 1993 values using same energy carrier prices for Slovakia.	
Finland			Total and Space heating Demand 1990 – 1994 for all energy carriers: Imputation using same data from Lee Schipper Database.
Germany	1990: Extrapolation from 1991 value using GDP time series		
Greece	1990 – 1994: Extrapolation from 1995 value using GDP time series	Price gas 1990 – 1997: Extrapolation from 1998 value using oil prices.	
Hungary	1990 – 1994: Extrapolation from 1995 value using GDP time series	Price oil 1996 – 2004, 2008 – 2011: Imputation using price natural gas & Coal price 1997: Interpolation between 1996 and 1998 price.	
Ireland	1990 – 1998: Extrapolation from 1999 value using GDP time series	Price coal 1996 – 2008: Imputation using coal prices from Irish Energy Agency (SEAI).	
Italy		Price DH 1990 – 1998: Extrapolation from 1999 value using price of gas.	
Netherlands		Price DH 2003 – 2010: Interpolation between price of DH in 2002 and 2011.	
Poland	1990 – 1994: Extrapolation from 1995 value using GDP time series	Price oil 1990 – 1993: Extrapolation from 1994 value using price of coal (dominant energy carrier) & Price of dh 1990 – 1994: Estimated using price of coal (dominant energy carrier).	
Portugal	1990 – 1994: Extrapolation from 1995 value using GDP time series	Price oil and price gas 1990 – 1999: Extrapolation using price of oil in Spain.	Space heating demand 1990 – 1999 all energy carriers: Extrapolation using data from IEA Sankey charts & Average floor area 1996 – 2002: Interpolation between 1995 and 2003 values.
Romania		Price of oil 1990 – 2007: Extrapolation from 2008 value using price of gas (dominant carrier).	Space heating demand 1990 – 1991 all energy carriers: Extrapolation using data from IEA Sankey charts.
Slovakia		Price of oil 2008 – 2018: Extrapolation from 2007 value using price of gas (dominant energy carrier). Price of DH 1990 – 1993: Extrapolation from 1994 value using price of gas.	
Slovenia	1990 – 1994: Extrapolation from 1995 value using GDP time series	Price of oil 1997 – 2003: Imputation using price of oil in Croatia. Price of oil 1995 – 1997: Imputation using price of natural gas.	
Spain	1990 – 1994: Extrapolation from 1995 value using GDP time series		
UK	1990 – 1994: Extrapolation from 1995 value using earlier version of AMECO.		
All 28	2017 – 2018: Extrapolation from 2016 value using AMECO data category, Gross wages and salaries (D11) (UWSH) for 2016 – 2018.	Price of DH 2014 – 2018: Extrapolation using dominant energy carrier for each respective country where DH is used.	

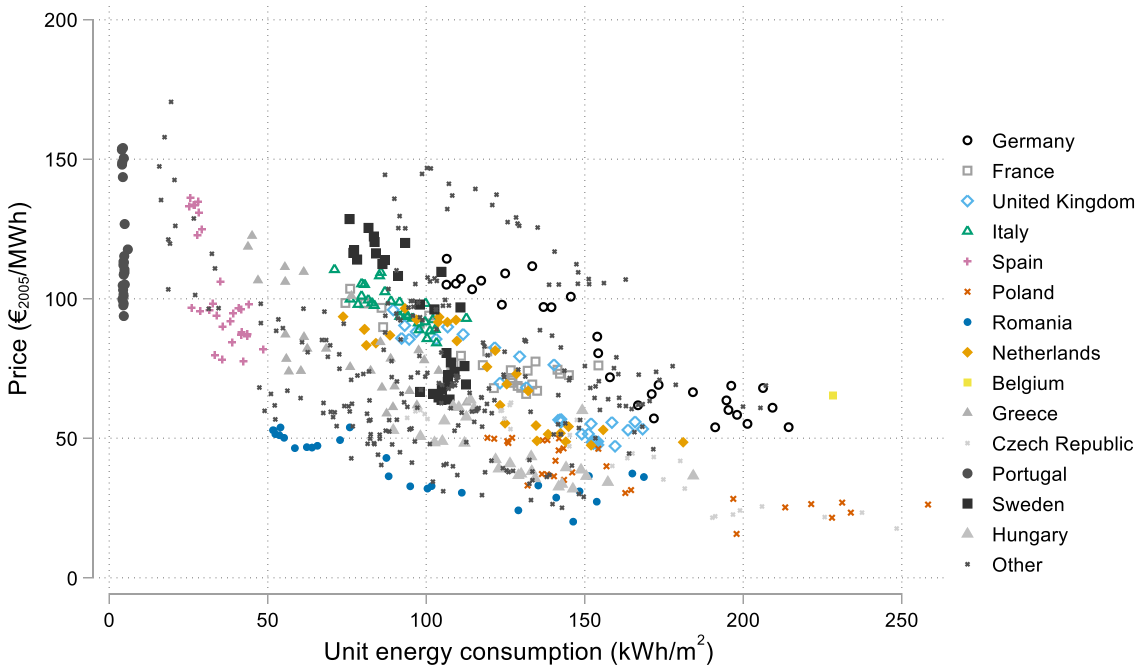

Figure S1. Residential energy for space heating – the demand curve.

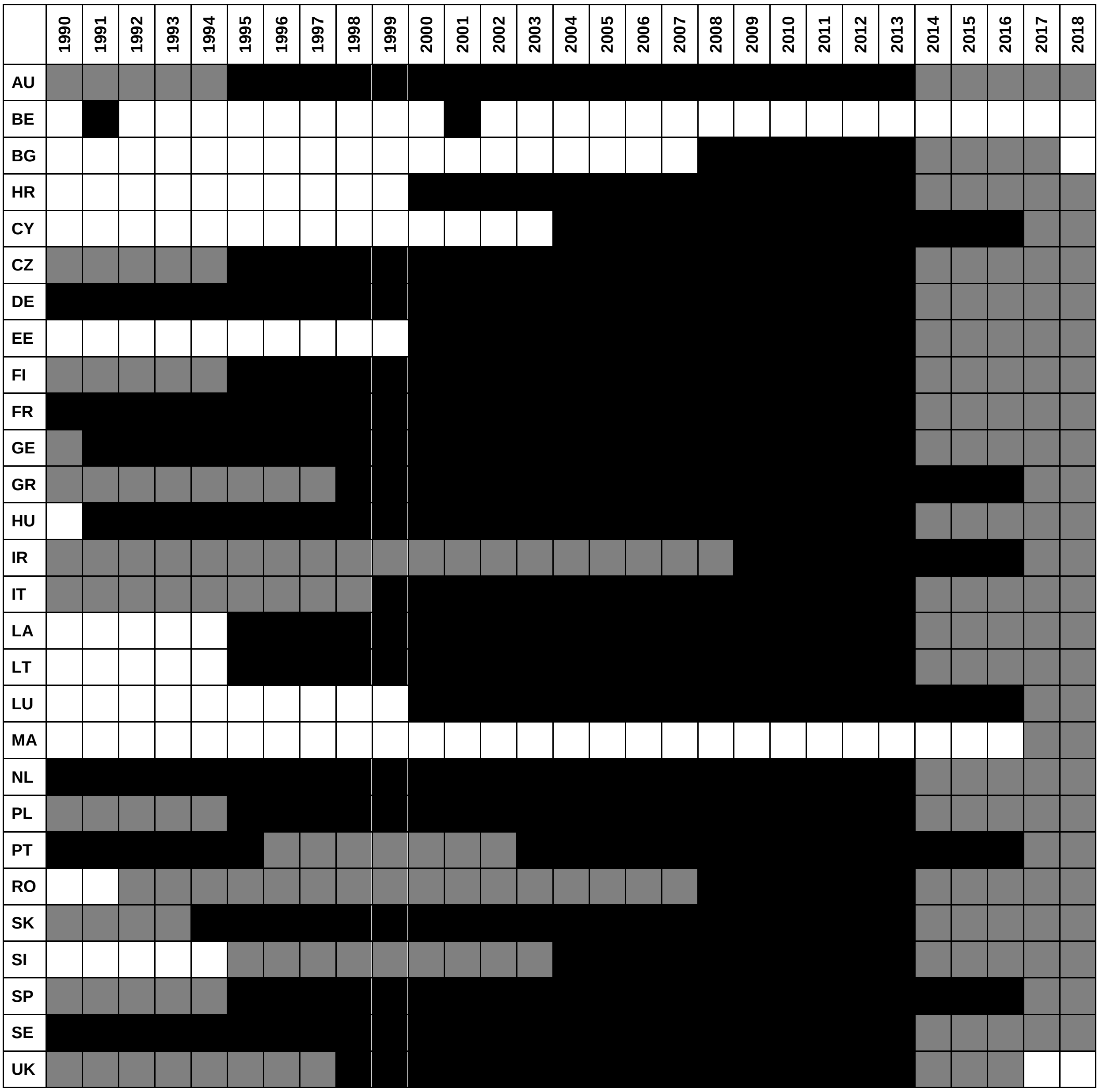

Figure S2. Data construction overview.

Black cells indicate where no data are missing for a country and year for any of the following five variables: total energy consumption, price, income, climate and floor area. Grey cells indicate where at least one of these five variables is imputed. White cells indicate where at least one of the five variables is missing. It is the white cells that ‘unbalance’ the panel.

Supplementary references

AMECO (2021) AMECO database. In: Eur. Comm. https://ec.europa.eu/info/business-economy-euro/indicators-statistics/economic-databases/macro-economic-database-ameco/. Accessed 1 Feb 2021

Enerdata (2021a) ODYSSEE database. http://odyssee.enerdata.net/database/. Accessed 1 Feb 2021

Enerdata (2020) Global Energy & CO2 Data. http://www.enerdata.net/brochure/Global-energy-market-CO2-data-brochure.pdf. Accessed 15 Nov 2020

Enerdata (2021b) MURE Database. http://www.measures-odyssee-mure.eu/. Accessed 1 Feb 2021

Eurostat (2021) Energy statistics - cooling and heating degree days. https://ec.europa.eu/eurostat/cache/metadata/en/nrg_chdd_esms.htm. Accessed 1 Feb 2019

Filippini M, Hunt LC, Zorić J (2014) Impact of energy policy instruments on the estimated level of underlying energy efficiency in the EU residential sector. Energy Policy 69:73–81. https://doi.org/10.1016/j.enpol.2014.01.047

Ó Broin E, Nässén J, Johnsson F (2015) Energy efficiency policies for space heating in EU countries: A panel data analysis for the period 1990-2010. Appl Energy 150:211–223

Werner S (2016) European District Heating Price Series
